# P-788. Exploring β-Lactam Interactions with DacB1: Unraveling Optimal Therapies for Combating Drug-Resistant *Mycobacterium tuberculosis*

**DOI:** 10.1093/ofid/ofae631.982

**Published:** 2025-01-29

**Authors:** Mary Nantongo, David C Nguyen, Eunjeong Shin, Christopher Bethel, Magdalena A Taracila, Khalid M Dousa, Sebastian G Kurz, Liem Nguyen, Barry N Kreiswirth, Wilem Boom, Robert A Bonomo

**Affiliations:** Case Western Reserve University, Cleveland, Ohio; Rush University Medical center, Chicago, Illinois; Case Western Reserve University, Cleveland, Ohio; Louis Stokes Cleveland VA Medical Center, Cleveland, Ohio; Case Western Reserve University, Cleveland, Ohio; VAMC, Cleveland, Ohio; University Hospitals of Tuebingen, Tuebingen, Baden-Wurttemberg, Germany; Case Western Reserve University, Cleveland, Ohio; Center for Discovery and Innovation, Hakensack Meridian Health, Nutley, New Jersey; Case Western Reserve University/ University Hospitals Cleveland Medical Center, Cleveland, Ohio; Case Western Reserve University/ Louis Stokes Cleveland VA Medical Center, Cleveland, OH

## Abstract

**Background:**

*Mtb* is a global health problem. DacB1, a D, D-carboxypeptidase, involved in *Mtb* peptidoglycan biosynthesis, is a promising target for β-lactam antibiotics (BLs) underutilized in *Mtb* treatment. Dual BL therapy may inactivate multiple targets in the peptidoglycan synthesis pathway (“target redundancy”). Modeling studies with meropenem (MEM), showed that the active site of DacB1 encompasses three penicillin-binding protein motifs: S121XXK124, S176XN178, and K282TG284 (PDB 4PPR). We explored the structure-activity relations, SAR, between DacB1 and other BLs to gain insight into optimal inhibitors for this important drug target.

**Figure:**
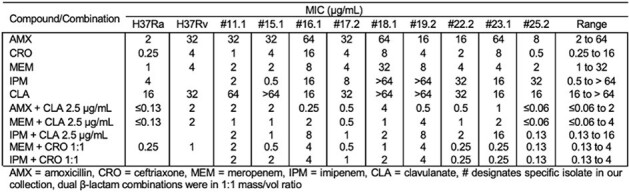

Tab. 1: β-lactam and β-lactamase Inhibitor MICs for Mtb H37Rv, H37Ra and representative Clinical Isolates

**Methods:**

Minimum inhibitory concentrations (MICs) of BLs and β-lactamase inhibitors (BLIs) against *Mtb* H37Ra, H37Rv, and representative clinical isolates were tested via broth microdilution. Timed electrospray ionization mass spectrometry captured acyl-enzyme adducts of DacB1 and BLs [amoxicillin (AMX), ceftriaxone (CRO), tebipenem (TBP), imipenem (IPM), MEM] or BLIs [clavulanate (CLA), sulbactam (SUL)]. Molecular docking analyses of MEM, IPM, TBP, and Michaelis-Menten complexes, were conducted.

**Fig.1:**
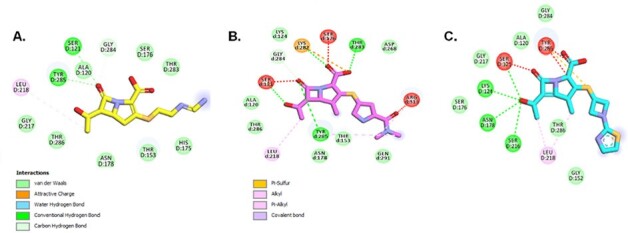
The 2D representation of Michaelis-Menten complexes of DacB1 and IPM (A.), MEM (B.), and TPB (C.) with the formation of initial interactions. The carbonyl group of all the carbapenems is positioned into the oxyanion hole fromed by the catalytic Ser121:N and by Tyr285 amide. the hydroxyethyl group on the β-lactam ring present on all carbapenes produce a hydrophobic interaction with Leu218 and helps the initial positioning od the carbonyl into the oxyanion hole of DacB1. The C1 methyl group in both MEM and TBP, with additional hydrophobic interactions (B. and C.), contribute to the slower acyl-enzyme formation fro those compounds.

**Results:**

MICs for IPM, MEM, and CRO ranged from 0.5 → >64, 1 → 32, and 0.25 → 16 µg/mL respectively (***Tab. 1***). Combining IPM or MEM with CRO further lowered MICs up to 32-fold (0.13 → 4 µg/mL), all within clinically achievable concentrations. Acyl-enzyme adducts of DacB1 (MW = 34414 Da) with carbapenems were captured; but not with AMX, CRO, or BLIs even after 120 min incubation. DacB1-IPM complexes were detected with 5 min incubation (Δ+299 Da) while at 15 min and 30 min for -MEM (Δ+383 Da) and -TBP (Δ+384 Da) complexes respectively.

**Conclusion:**

IPM preferentially binds to DacB1 compared to MEM and TBP, and BLs, shedding light on crucial SAR for drug development. We postulate that the carbonyl group present in all carbapenems assists with positioning into the oxyanion hole of DacB1. However, the C1 methyl in MEM and TBP with their additional hydrophobic interactions contribute to steric hindrance and slower acyl-enzyme formation (***Fig. 1***). This study advances our understanding of the molecular mechanisms governing DacB1 interactions with BLs, offering insights into how carbapenems may inhibit another important BL target in *Mtb* peptidoglycan synthesis.

**Disclosures:**

**All Authors**: No reported disclosures

